# Aggressive Angiomyxoma in the Scrotum: A Case Series and Literature Review

**DOI:** 10.3389/fsurg.2022.762212

**Published:** 2022-03-02

**Authors:** Juan Sun, Peng H. Lian, Zi X. Ye, De X. Dong, Zhi G. Ji, Jin Wen, Han Z. Li

**Affiliations:** ^1^Division of General Surgery, Department of Surgery, Peking Union Medical College Hospital, Peking Union Medical College and Chinese Academy of Medical Sciences, Beijing, China; ^2^Division of Urology, Department of Surgery, Peking Union Medical College Hospital, Peking Union Medical College and Chinese Academy of Medical Sciences, Beijing, China

**Keywords:** aggressive angiomyxoma, scrotum, benign tumor, surgical excision, local recurrence

## Abstract

**Purpose:**

Aggressive angiomyxoma (AAM) was identified as a distinct clinicopathological entity in 1983. Since then, a few cases of its occurrence in the scrotum have been reported. This case series was performed to increase clinicians' understanding of the clinical features and treatment of AAM in the scrotum.

**Methods:**

We evaluated the clinical presentations, treatments, and follow-up of two patients with AAM in the scrotum in our hospital and 34 cases reported in the literature.

**Results:**

Among the 36 patients, the average age was 48.3 ± 20.6 years old (range from 1 to 81); the average maximum diameter of the tumor was 8.36 cm (1.6–25 cm); the site of one (2.78%) patient was located in the epididymis, two (5.56%) in the testes, five (13.89%) in the spermatic cord, and 28 (77.77%) in the scrotum. The clinical symptoms were generally non-specific and 20 patients inadvertently discovered their slow-growing painless masses. The treatments for all these patients were surgical excision once the tumor had been found and one case underwent excision followed by radiotherapy. The median follow-up time for the remaining 32 cases was 24.5 months (1 to 84 months). Recurrence occurred in three cases (9.09%) at the primary sites and no cases of distant metastasis.

**Conclusion:**

AAM of the scrotum can occur in middle-aged and elderly men. The clinical manifestation generally involves a long history of asymptomatic masses or swelling in the scrotum. Ultrasound is the most commonly used diagnostic technique but magnetic resonance imaging may be more effective. The mainly treatment is surgical excision and postoperative histopathological examination is still the gold standard for its diagnosis. Although it is locally aggressive, metastasis is extremely rare in males.

## Introduction

Aggressive angiomyxoma (AAM) is a rare benign mesenchymal myxoid tumor that usually occurs in the pelvic soft tissues and perineum of women in the reproductive age (1). It was known for its invasiveness, recurrence and slow growth (2). AAM rarely affects male and non-pelvic-perineal anatomical sites, so few reports can be found on AAM in men and much fewer on the scrotum. More specifically, the incidence of AAM in females is about six-fold higher than in males and the age distribution is wide, ranging from 6 to 77 years (3). AAM is often characterized by painless masses, which making it hidden and difficult to detect. It is usually treated when the mass is large or has compression symptoms (4, 5). Although radiological techniques such as ultrasound (US), computed tomography (CT), and magnetic resonance imaging (MRI) are helpful for reaching a diagnosis, the gold standard diagnostic method is still histopathological examination (6). Histologically, AAM exhibits a low to moderate cellularity and is composed by a population of uniform, spindled cells featuring a low mitotic count (7). Surgery is the first line and the most important method for the treatment of it. However, half of the patients appeared local recurrence even after complete resection but distant metastasis is rare (5).

At present, AAM-related literature mainly consists of studies about women and a few case reports in men, lacking systematic analysis and summary of a certain number of cases in terms of AAM in the scrotum. To help recognize AAM occurred in scrotum better and reduce the rate of misdiagnosis, we herein describe two cases of it in Peking Union Medical College Hospital (PUMCH) and further review 34 cases in literature provides a retrospective analysis of their clinical manifestations, diagnosis, and treatments of this condition.

## Materials and Methods

Between January 1983 and December 2020, a total of 2 patients with AAM in the scrotum were diagnosed and treated at the PUMCH. The medical histories and preoperative auxiliary examinations were taken from medical records, including tumor marker test, US and MRI. Histopathological and immunohistochemical characteristics were also reviewed. The two cases underwent individualized surgical treatment and were diagnosed by postoperative paraffin pathological examination.

PubMed and Geen Medical databases were searched for clinical case reports published from 1992 to 2020 using the keywords of aggressive angiomyxoma and scrotum. The included cases had full clinical data and clear pathological diagnosis. Finally, 34 cases met the inclusion criteria, including the two cases in our hospital, there were 36 cases included in total.

Observed indexes: The ages, localization and sizes of the tumors, symptoms and duration, therapies and follow-up were documented and retrospectively analyzed.

## Case Reports

### Case 1

**Figure 1:**
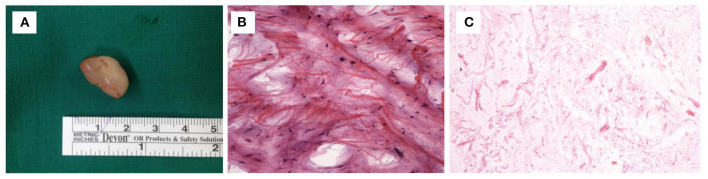
**(A)** Gross appearance of AAM in Case 1. **(B)** Intraoperative frozen section in Case 1 showing mass polygonal cells and fat cells. (HE × 200). **(C)** Postoperative histopathology in Case 1 showing scattered blood vessels and fibroblasts with the background of mucus (HE × 40).

Case 1 was a 45-year-old male who was admitted to our hospital with a one-year history of a growing left scrotal mass. Physical examination on arrival at our hospital revealed a circular lump of about 1.5 cm in diameter palpated on the left side of the scrotum with no pain but mild tenderness. The mass had a clear boundary, moderate mobility, and normal overlying skin. The light transmittance test was negative, and the remaining physical examination and laboratory test results, including routine blood examination, liver and kidney function tests, blood coagulation parameters, and tumor markers [alpha fetoprotein, carcinoembryonic antigen, cancer antigen (CA) 19-9, CA242, CA125, and CA72-4], were unremarkable. The US of the scrotum showed that a 1.6 × 1.2 × 1.0 cm area of clear boundary and low echo could be seen on the left spermatic cord with a meager blood flow signal on color Doppler flow imaging (CDFI). On suspected lipoma of the spermatic cord, the patient underwent a tumor resection on his fifth day in the hospital. Intraoperatively, the mass was found to be approximately 1.2 × 1.0 cm (Figure 1A) and beside the vas deferens. The intraoperative frozen section showed that the tumor originated from mesenchymal tissue (Figure 1B), whereas the postoperative pathology showed it was an angiomyxoma (Figure 1C). During the 6-year follow-up, the patient showed no recurrence.

### Case 2

**Figure 2:**
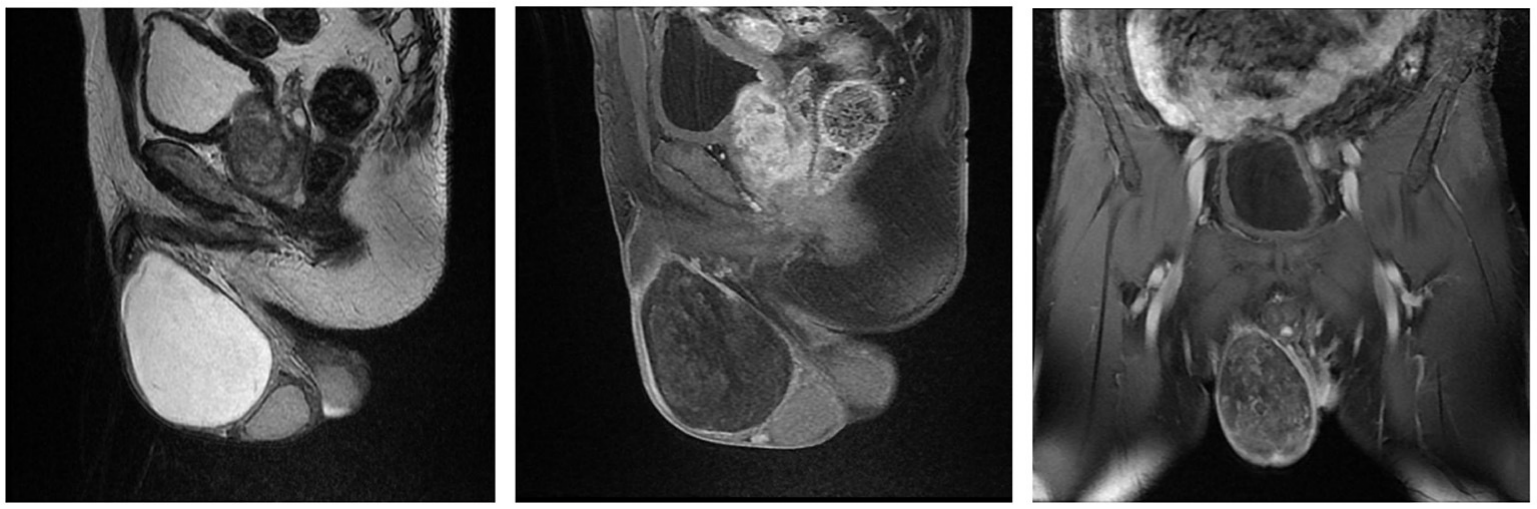
Magnetic Resonance Imaging of Case 2 showing that a size of 9.6 × 6.9 × 6.2 cm elliptical long T1 and long T2 signal mass in the right scrotum, which presented as an iso-height signal on DWI sequence and small spots of enhancement on the enhanced scan.

Case 2 was a 64-year-old man who was admitted to our urology department on March 20, 2015 with a seven-month history of a right scrotal mass accompanied by obvious plummeting. Physical examination on arrival at our hospital revealed an enlarged right scrotum, nearly 10 cm in diameter with no pain or tenderness, and the scrotum did not disappear while lying flat. The light transmittance test was negative, and the other physical examination and laboratory test results were all unremarkable. The US of the scrotum revealed that a gelatinous uneven echo could be seen at the right spermatic cord and the upper part of the scrotum, and CDFI showed negligible blood flow signal. MRI showed an elliptical long T1 and long T2 signal mass in the right scrotum, with a clear boundary and size of about 9.6 × 6.9 × 6.2 cm, which presented as an iso-height signal on the diffusion weighted imaging sequence and small spots of enhancement on the enhanced scan (Figure 2). Finally, right scrotal mass resection was performed and the postoperative histopathological examination showed AAM of the scrotum. The immunohistochemistry showed: CD 31 (+), CD 34 (+), S-100 (+), BCL-2 (–), SMA (–), and Ki-67 (index 6%). During the 6-year follow-up, the patient showed no recurrence.

## Results

Among these 36 patients, the average age was 48.3 ± 20.6 years old (range from 1 to 81) when AAM was diagnosed, and detailly five patients (13.89%) were <20 years old, 3 (8.33%) were 8 20–40 years old, 24 (66.67%) were 40–70 years old, and 4 (11.11%) 9 were >70 years old. With respect to the location of the tumor, one (2.78%) was in the epididymis, two (5.56%) in the testes, five (13.89%) in the spermatic cord, and the remaining 28 (77.77%) were reported in the scrotum. The average maximum diameter of the tumor was 8.36, while the minimum was 1.6, the maximum was 25, and 10 patients had tumors larger than 10 cm in size (28.6%) (Table 1).

**Table 1:** Reported cases of AAM in the scrotum from literature.

	**Age**	**Location**	**Size (cm)**	**Symptoms and duration**	**Therapy**	**Follow-up**
1	42	Left scrotum	5 × 3 × 1 cm	A noticed mass, 2 mo	Wide excision	NED after 3 y (8)
2	70	Spermatic cord	14 × 6 × 3 cm	A noticed mass, 3mo	Simple excision	NED after 6 y (8)
3	58	Left scrotum	14 × 8 × 6 cm	Painless mass, 12 mo	Local excision; radiation therapy	NED after 25 mo (8)
4	45	Left scrotum	3.4 × 2.6 × 1.8 cm	Painless mass, unspecified duration	Local excision	NED after 18 mo (9)
5	62	Midline scrotum	6 × 4 × 2 cm	Painless mass, unspecified duration	Local excision	NA (9)
6	51	Inferior scrotum	4.5 cm in greatest dimension	Painless mass, 4 weeks	Local excision	Local recurrence at 6.2 years (9)
7	61	Left scrotum	25 × 16 × 15 cm	A noticed swelling, 4 years	Local resection	NED after 11 mo (10)
8	77	Left spermatic cord	6 × 6 × 6 cm	Circumscribed mass, NA	Radical orchiectomy	Multiple recurrences after 7 y (11)
9	54	Right scrotum	5 × 5 × 2.5 cm	Slowly enlarging scrotal mass, 4 mo	Local excision	NED after 28 mo (12)
10	37	Left scrotum	7 × 5 × 4 cm	Hydrocele, NA	Local excision	NED after 2 y (13)
11	26	Left scrotum	7.5 × 6.0 × 5.0 cm	Slowly enlarged mass, 2.5 y	Surgical excision	NED after 7 mo (14)
12	13	Left spermatic cord	9.5 × 4 × 2.2 cm	Pain less subcutaneous cyst,	Surgical excision	NED after 30 mo (15)
13	13	Right scrotum	2 × 1.7 × 1.4 cm	Swelling, NA	Surgical excision	NA (15)
14	24	Right scrotum	7 × 5 × 5 cm	An increasing scrotal mass, 1 y	Wide excision	NED after 26 mo (16)
15	8	Left scrotum	1.7 × 0.7 × 0.6 cm	Asymptomatic scrotal mass, NA	Surgical enucleation	NED after 1 y (17)
16	1	Scrotum	17 × 11 × 4 cm	Rapidly enlarging tumor, 100 days after birth	Surgical excision	NED after 3 mo (17)
17	47	Right scrotum	17 × 16 × 6 cm	Slowly enlarged mass, 2 y	Surgical excision	NED after 14 mo (1)
18	57	Scrotum	5 cm	NA	Wide Surgical excision	NED after 72 mo (18)
19	44	Right epididymis	8 cm	NA	Surgical excision	NED after 12 mo (18)
20	69	Testis	6 cm	NA	Wide excision	NED after 1 mo (18)
21	43	Left scrotum	4.5 × 3.5 × 1.5 cm	A painless scrotal mass, NA	Surgical excision	NED after 41 mo (19)
22	68	Left scrotum	5.5 × 4.5 × 4 cm	An asymptomatic testicular mass, 1 mo	Wide surgical excision	NED after 34 mo (19)
23	81	Spermatic cord	2 × 1 × 1 cm	Inguinal hernia, NA	Surgical excision	NED after 32 mo (19)
24	48	Left spermatic cord	6 × 5 × 3.5 cm	A left scrotal mass, NA	Surgical excision	NED after 32 mo (19)
25	12	Right testicle	4.5 × 3.5 × 0.5 cm	Slow enlargement of right testicle	Surgical excision	Recurrence after 2 mo (20)
26	40	Right scrotum	5.0 × 5.1 × 5.4 cm	Slowly grow mass, 10 y	Surgical excision	NED after 12 mo (21)
27	64	Median scrotum	7 × 11 × 19 cm	Hydrocele	Surgical excision	NED after 3 y (22)
28	40	Left scrotum	5.1 × 1.7 × 3.2 cm	A painless scrotal mass, NA	Surgical excision	NA (23)
29	62	Scrotum	15 × 12 cm	A painless scrotal swelling, 6 mo	Surgical excision	NED after 3 mo (24)
30	73	Scrotum	Estimated weight 5 kg	Monstrous scrotal lymphedema	Complete resection of the scrotal skin	NED, no interval was reported (25)
31	65	Right scrotum	3.4 × 3.8 × 4.8 cm	Swelling and enlarging in size, 6 mo	Radical right orchidectomy	NED after 2 y (26)
32	66	Right scrotum	11.5 × 7.5 × 7 cm	Slowly growing mass, 20 y	Right orchiectomy	NED after 6 mo (27)
33	53	Left scrotum	16.5 × 12.0 cm	Slowly growing swelling, 3 y	High orchiectomy	NED after 12 mo (28)
34	57	Scrotum	11 cm	a feeling of heaviness at the scrotum	Surgical excision	NED after 2 y (29)
35	45	Left scrotum	1.6 × 1.2 × 1.0 cm	A painless scrotal mass, 1 y	Surgical excision	NED after 6 y (our first case)
36	64	Right scrotum	9.6 × 6.9 × 6.2 cm	A mass with obvious plummeting, 7 mo	Surgical excision	NED after 6 y (our second case)

*NED, no evidence of disease; NA, information not available*.

Patients' clinical symptoms were generally non-specific. There were 20 patients who inadvertently discovered their slow-growing painless masses. The remaining patients experienced clinical symptoms such as swelling in the scrotum, hydrocele, inguinal hernia, lymphedema, and so on. And the range time from tumor discovery to treatment was 1 months to 20 years.

In our report, all of the 36 cases underwent surgical excision once the tumor had been found and one of them underwent excision followed by radiotherapy. For 27 cases, local surgical excision was performed to remove the tumors while wide surgical excision was done for four cases. In one case, the patient operated with complete resection of the scrotal skin because of monstrous scrotal lymphedema. And radical orchiectomy was done in the rest of four cases.

Of these 36 cases, three patients were lost to follow-up and one case showed no interval for NED (no evidence of disease). The median follow-up time for the remaining 32 cases was 24.5 months (1 to 84 months). Recurrence occurred in three cases (9.09%), indicated by re-check of imaging examination. And the recurrence in all three patients was found at the primary sites and there were no cases of distant metastasis.

## Discussion

AAM or deep angiomyxoma is a rare and special type of soft tissue tumor that is recognized as a mesenchymal malignant tumor by the World Health Organization and was first described by Steeper and Rosai in 1983 (29). Aggressiveness refers to the nature of the tumor, local infiltration process, and frequent recurrence as opposed to its malignant potential (29). It rarely occurs in men, especially in the scrotum.

For the etiology of AAM, Kenny et al. found that the formation of new organisms in the lesion site of AAM may be associated with the loss of the X chromosome (30). Some authors believe that the invasiveness and growth pattern of AAM may be related to IL-5, PGF-R and other genes that are activated by the translocation or insertion of 5q31 and 12p11 (31). However, at present its pathogenesis is still unclear.

Most patients with AAM present with a long history of asymptomatic masses or swelling in the scrotum. In our 36 cases, the size of the masses ranged from 1.6 to 25 cm and the history ranged from 1 month to 20 years. The private location and painless symptoms led to delayed medical treatment, resulting in a gradually increased tumor size and high clinical misdiagnosis rate. Clear preoperative diagnosis is difficult, and differential diagnosis includes non-neoplastic causes, such as inguinal hernia, edema, testicular, paratesticular tumors (28). It also needs to be differentiated from other mesenchymal tumors in this region, such as angiomyofibroblastoma, cellular angiofibroma, and superficial angiomyxoma (5).

In terms of the preoperative diagnosis of AAM in the scrotum, the currently used auxiliary examinations mainly include US, CT, and MRI (32). AAM lesions usually show as irregular hypoechoic masses on US that involve a large range and relatively clear edges. Some AAMs presented as heterogeneous mixed hypoechoic masses, which may due to different tissue density and interstitial edema, as well as local mucoid degeneration or cystic change. CDFI was able to detect a rich blood flow signal inside and around the tumor, which may be associated with characteristic vascular components of AAM (33). On a plain CT scan, it is expressed as a low-density shadow relative to the muscle tissue and swirling or stratified mild enhancement on enhanced CT (34). When an AAM is viewed on MRI, the T1-weighted phase appears with low or intermediate signal intensity whereas the T2-weighted phase often shows high signal intensity, which can be used to differentiate other soft tissue tumors (34). MRI is more specific than US and CT, which is of great significance to clarify the tumor scope and its relationship with surrounding tissues and organs. Besides, tumor biomarkers are usually normal, preoperative fine-needle aspiration cytology seems to be helpful but the puncture area may be blood vessel rich and bleeding caused by puncture may prevent discovery of tumor cells (35). From the above, non-specific tumor biomarkers, imaging and intraoperative pathology examinations can only assist the diagnosis, but the gold standard for diagnosis of AAM relies on the histopathological examination of postoperative specimens.

On gross evaluation, AAM is usually a partially limited, uneven, lobulated, soft, and grayish-white tumor with a smooth contour and colloid appearance. Histologically, nearly all AAMs are identical, and characterized by prominent vascular components and invasive growth. They are composed of thick- or thin-walled vessels of varying sizes, and the tumor cells are evenly distributed in the stroma-containing mucus, showing as uniform spindled, star shaped, or oval shaped. Further, bland nuclei and sparse mitoses are its cytological characteristics, and nuclei with morphological changes such as abnormal shape and nuclear division are rare (36). So there were studies speculated that AAM may originate from stem cells with multi-differentiation potential around the blood vessels that differentiate into fibroblasts and myofibroblasts (37).

Immunohistochemically, AAM is positive for Desmin, Vimentin, SMA (smooth muscle actin) and CD34, and negative for S-100, CK and CD68 (38). Among them, the positive Desmin and SMA are helpful for diagnosis due to non-specific gene-level characteristics. Besides, Ki-67 is related to the activity of tumor cell proliferation, the lower Ki-67 level, the lower proliferative activity. Estrogen receptor and progesterone receptor were positive in most female AAM patients, suggesting that hormone effect may be involved in the tumor (39), but it is not applicable in AAM in the scrotum. In general, AAM can be diagnosed by hematoxylin staining, but immunohistochemistry can provide important evidence for differential diagnosis.

The differential diagnosis of AAM with other tumors mainly contains angiomyofibroblastoma (presents as thin-walled blood vessels and perivascular cuffing by bland-appearing stellate, areas of marked hyalinization and spindled and plasmacytoid cells, as well as well-circumscribed or encapsulated tumors), myxoid neurofibroma (commonly occurs in extremities and is strongly positive for S-100), myxoma (usually occurs in the muscles of the extremities and are fibroblastic in nature), myxofibrosarcoma (a malignant tumor in which mitotic activity is usually readily identified), myxoid liposarcoma (univacuolated lipoblasts and abundant thin-walled vessels) (19). Other differential diagnosis includes perineal cyst, inguinal hernia, hydrocele, abscess which can be distinguished from AAM by US or other auxiliary examinations (29).

The mainstay treatment of AAM in the scrotum is wide local excision with tumor-free surgical margins (40). However, A study including 106 patients with AAM showed that there were no significant differences in long-term recurrence (10 years) rates between positive and negative margins (40 vs. 50%) (41). And because of just local recurrence and almost no metastasis tendency of AAM, it can still have secondary resection once recurrence occurred. Some experts believe that incomplete or partial resection is acceptable when the incidence of surgical complications is high or fertility retention becomes a problem (41, 42).

The risk of local recurrence of AAM is high (35 to 72%), especially within 2–3 years after the first operation (43). Approximately 50% of recurrences occur within the first five years after surgery (25). There has been no report of distant metastasis in male patients with AAM, but two cases of lung and mediastinal metastasis in females have been reported (44, 45). The recurrence rate varies with gender (female: 46, male: 9%) (23). Due to the risk of late recurrence, close follow-up is still required. It has been proposed to follow up each year by means of imaging and to conduct regular clinical supervision (24).

As for those AAM patients of local recurrence, radiotherapy has been proposed as a control method for multiple recurrence after surgical resection, but the effect is not satisfactory; and chemotherapy is often ineffective because of low mitotic activity of AAM (46). Vascular embolization can diminish tumors by blocking their blood supply. It can also reduce intraoperative bleeding and operation risk (47). But the tumor blood supply may come from multiple arteries, so the rapid development of alternative blood supply in tumor after embolization may lead to the recurrence. Therefore, it is difficult to completely destroy tumors by embolization alone, which is usually used for surgical adjuvant therapy (42, 48). So long-term imaging follow-up is still essential regardless of treatment due to high AAM recurrence rate.

In conclusion, AAM of the scrotum can occur in middle-aged and elderly men. The clinical manifestation generally involves a long history of asymptomatic masses or swelling in the scrotum. US is the most commonly used diagnostic technique preoperatively but MRI may be more effective. The mainly treatment is surgical excision and postoperative histopathological examination is still the gold standard for its diagnosis. Although it is locally aggressive, metastasis is extremely rare in males.

## Data Availability Statement

The original contributions presented in the study are included in the article, further inquiries can be directed to the corresponding author/s.

## Author Contributions

JS and PL were responsible for collecting, sorting out data and writing articles, were co-first authors. ZY, DD, and ZJ were responsible for collecting data. JW and HL were responsible for putting forward ideas and reviewing articles, were co-corresponding authors of this paper.

## Funding

Non-profit Central Research Institute Fund of Chinese Academy of Medical Sciences (2019XK320027) and Project Management Fund for Foreign Cultural and Educational experts (G20190001645).

## Conflict of Interest

The authors declare that the research was conducted in the absence of any commercial or financial relationships that could be construed as a potential conflict of interest.

## Publisher's Note

All claims expressed in this article are solely those of the authors and do not necessarily represent those of their affiliated organizations, or those of the publisher, the editors and the reviewers. Any product that may be evaluated in this article, or claim that may be made by its manufacturer, is not guaranteed or endorsed by the publisher.
